# Control of *Nematodirus* spp. infection by sheep flock owners in Northern Ireland

**DOI:** 10.1186/s13620-017-0109-6

**Published:** 2017-10-19

**Authors:** Connor McMahon, Hillary W. J. Edgar, Jason P. Barley, Robert E. B. Hanna, Gerard P. Brennan, Ian Fairweather

**Affiliations:** 1 Parasite Therapeutics Research Group, School of Biological Sciences, Medical Biology Centre, The Queen’s University of Belfast, 97 Lisburn Road, Belfast, BT9 7BL UK; 2 Veterinary Sciences Division, Agri-Food and Biosciences Institute (AFBI) Stormont, Belfast, BT4 3SD UK

**Keywords:** *Nematodirus* spp., Survey, Questionnaire, Northern Ireland, Faecal egg count reduction test, Egg hatch behaviour

## Abstract

**Background:**

To address a lack of information on the control of ovine helminth parasites in Northern Ireland (NI), a number of research projects have been undertaken, dealing with gastrointestinal nematodes, tapeworms and liver fluke. This investigation concerns *Nematodirus* and concentrates on three aspects of disease: farm management strategies for its control, derived from the results of a Questionnaire; the efficacy of treatment used by farmers, as determined by a coprological survey; and the hatching requirements of *Nematodirus* eggs, that is, whether prolonged chilling is a pre-requisite for hatching.

**Results:**

A Questionnaire was sent to 252 sheep farmers in NI in March 2012 (covering the years 2009–2012) and replies were received from 228 farmers. Under-dosing, inaccurate calibration of equipment and inappropriate product choice were poor practices identified. Following this survey, the efficacy of treatment of *Nematodirus* spp. in sheep flocks was evaluated in April and May 2012. Sampling kits were sent to 51 flock owners, all of whom returned pre- and post-anthelmintic dosing faecal samples to the laboratory for analysis. At the time of treatment, 41 flocks were positive for *Nematodirus* (as diagnosed by the presence of eggs). Reduced benzimidazole efficacy was detected in 35.7% of flocks tested (*n* = 28). Although only involving a small number of flocks, reduced efficacy of levamisole treatment was detected in 50%, of avermectins in 33% and of moxidectin in 75% of flocks tested (n = 2, 6 and 4, respectively). In the egg hatch experiment, carried out under “chilled” and “non-chilled” conditions, 43% of the eggs in the “non-chilled” group were able to hatch, compared to 100% in the “chilled” group.

**Conclusions:**

The identification of inefficient control strategies argues for continued education of stockholders, in order to improve their management programmes. This is particularly important where the practices might impact on the development of anthelmintic resistance, which has been shown to exist on NI farms. The appropriate choice of anthelmintic is a vital part of this plan. The ability of eggs to hatch under non-chilled conditions demonstrates a flexibility in hatching behaviour. This may represent an adaptation to climate change and account for the recent emergence of a second, autumnal peak of infection.

## Background

In relation to published data on the control of helminth parasite disease in livestock, historically, Northern Ireland (NI) has lagged behind the rest of the United Kingdom (UK) and the Republic of Ireland (ROI). There is little information on topics such as the epidemiology (and changing pattern) of disease, the prevalence of disease, the level of anthelmintic resistance (AR) in parasite populations and what management strategies are in place to control disease. This is surprising, given that the NI economy is more dependent upon agriculture than any other region of the UK, as seen through its share of the economy, employment and business base [1]. The gross value added (GVA) of the combined Agriculture and food/drink processing industry was 3.5% to the GVA for NI, and employment was 6% [2]. Beef and sheep meat encompass the largest sector of the Agri-Food industry: £394 m and £63 m was accrued from finished cattle and finished sheep in 2015, respectively [3]. The gathering of data in relation to parasitic diseases is of vital importance to the design and development of effective and sustainable programmes for parasite control. That is, programmes that will enable farmers to maximise animal welfare and productivity, yet will mitigate dependence on drug use, and thereby serve to slow the progression of AR.

The results of some recent studies on ovine helminth parasites in NI have begun to fill in the gaps in our knowledge. Climate change has been shown to have altered the seasonal pattern of some diseases: for example, an extension of the traditional transmission window for trichostrongylosis/teladorsagiosis; the emergence of a second, autumnal peak in *Nematodirus* spp. infection; and a shift in chronic fasciolosis to earlier in the year [4–6]. A coprological survey carried out in July–October 2011 provided information on the prevalence of AR in gastrointestinal nematode infections in sheep [7]. A Questionnaire survey (conducted between May–September 2011) examined patterns of anthelmintic drug use and the effectiveness of management strategies in place to control the diseases [8, 9]. However, the timing of the coprological survey in 2011 precluded the collection of data in relation to *Nematodirus* spp.

In sheep flocks in the UK and elsewhere in Europe, also Canada and the Rocky Mountain States of the USA, the most common aetiological agent of nematodirosis is *Nematodirus battus* [10]. While *Nematodirus helvetianus, N. filicollis* and *N. spathiger* have been noted in mixed infections in these areas, they are considered to be more common across Australasia. *Nematodirus* spp. are considered to be atypical among trichostrongylid nematodes as a result of their long generation time, development to L_3_ larvae within the egg and (presumed) requirement of a period of chilled temperatures, followed by a sustained daily average temperature of 10 °C or more before hatching will take place [11, 12]. Nematodirosis is predominantly a disease of young lambs in the Spring; immunity develops quickly [13] and this results in adult stock playing a negligible role in epidemiology [14]. *N. battus* infection is an important cause of clinical disease (resulting from scouring and reduced weight gain) and fatality in young lambs in spring, following the mass hatch of infective L_3_ larvae [15].

Relatively little is known about nematodirosis in NI. Diagnoses of nematodirosis (as a percentage of all ovine submissions to the Agri-Food and Biosciences Institute (AFBI) in NI and The Department of Agriculture, Food and the Marine (DAFM) Laboratories in the ROI) were 16%, 11% and 16% higher in NI than the RoI in 2013, 2014 and 2015, respectively [16–18]. In terms of the percentage of all ovine endoparasitic disease, nematodirosis in NI stands at 22%, compared to 27% for PGE (caused by other trichostrongylid nematodes); for the ROI, the figures are 6% and 52%, respectively [18]. The data suggests that nematodirosis is more prevalent in the North of Ireland than in the South.

It has been shown that up to ~70% of all deposited eggs will hatch without the chilling stimulus [19] and it has been suggested that the phenotypic plasticity in *N. battus* hatching behaviour represents a “bet-hedging” strategy that allows the establishment of “chilled” larvae in parasite-naïve lambs, supplemented by infection later in the season by larvae produced from “non-chilled” eggs [20]. In recent years, increasingly, outbreaks of nematodirosis have been seen later in the grazing season (or indeed in the Autumn) in older lambs [20–27]. This has already been reported for NI [4].

Control of nematodirosis is achieved through the use of anthelmintic drugs, most commonly benzimidazoles (BZs). In contrast to the situation in other trichostrongylid nematodes, there have been relatively few reports of AR in *Nematodirus* spp. In the Southern Hemisphere, oxfendazole resistance was found on a sheep farm in New Zealand [28], and *Nematodirus* spp. were found to be resistant to oxfendazole, thiabendazole and fenbendazole in a survey conducted on sheep farms in Australia [29]. Furthermore, a total of 8 isolates of BZ-resistant *N. spathiger* (with a small contribution to total parasite burden by *N. abnormalis*) were reported in Tasmania [30, 31]. More recently, BZ (albendazole) resistance has been demonstrated in *N. spathiger* and *N. filicollis* in New Zealand [32]. In the Northern Hemisphere, anthelmintic failure against *N. battus* has been reported in the UK and the ROI; in the UK, it was linked with (the impact of) “intestinal hypermotility on the pharmacokinetics of relatively insoluble drugs”, rather than to AR *per se* [33]. Similarly, evidence for reduced efficacy of levamisole (LV) and macrocyclic lactone (ML) treatments has been observed in the ROI, with suboptimal dosing practices put forward as a potential explanation [34, 35]. Recently, a fenbendazole-resistant *N. battus* isolate was identified in Scotland [36]. A subsequent clinical efficacy trial and pyrosequencing analysis revealed a high frequency of homozygous resistant genotypes, demonstrating that the F200Y Single Nucleotide Polymorphism (SNP) may be a potential mechanism of resistance in *Nematodirus* spp. [37].

The overall aim of the current investigation was to obtain more information on ovine nematode disease control in NI, by focussing on *Nematodirus*. The study examined three specific topics: the efficiency of management practices in use by farmers, as gathered from the results of a Questionnaire; the efficacy of treatment of *N. battus* populations, as determined by means of a coprological survey; and the hatching requirements of *N. battus* eggs, that is, whether chilling is essential for hatching of the eggs. The results of the latter experiment may shed light on the underlying cause of the changing pattern of infection in response to climate change, a second, autumnal peak of infection having been identified previously [4].

## Methods

### Questionnaire

Following the analysis of data collated from a Province-wide Questionnaire survey in 2011, a supplementary set of questions was sent (in March 2012) to those respondents who farmed sheep. The supplementary questions were presented in 3 sections, namely, control of *Nematodirus* spp. (Section 1), control of ectoparasites (Section 2) and control of tapeworm parasites (Section 3). This manuscript is only concerned with the responses to questions in Section 1.

### Statistical analysis of questionnaire data

Descriptive statistics, such as means and standard deviations, were calculated using Microsoft® Excel 2007.

### Coprological survey

#### Survey population

Through the completion and return of a Questionnaire, participants had indicated a willingness to have their flocks tested for the presence of anthelmintic-resistant *Nematodirus* spp. populations. On receipt of a completed questionnaire at the Veterinary Sciences Division (VSD), Stormont, flock owners were sent sample packs which contained a general overview of the test methodology. Following this, each flock owner was contacted by telephone to discuss the specific requirements of the testing protocol. No limitations were placed on the owners as to when they were to collect pre-treatment samples (regarding the time elapsed since the last anthelmintic treatment), or which product was to be used for treatment. Similarly, the only criterion for inclusion in the study was that a questionnaire was completed beforehand. No further selection of participants took place. The intent of this was to ensure inclusion of a diverse range of flock types, including pedigree, commercial and mixed flocks, as well as a range of flock sizes. Coprological testing took place between April 1st and May 31st 2012.

#### Instructions to flock owners

Calibration of dosing equipment (for products to be given orally) was to be performed, before treatment, using the drench of choice at its recommended dose rate. Flock owners were to dispense 20 ml of anthelmintic solution into a graduated measuring device, to ensure the appropriate volume was being delivered. To minimise under-dosing, flock owners were asked to weigh the animals (by weigh-bridge) and to dose to the weight of the heaviest animal in the group, as per SCOPS (Sustainable Control Of Parasites in Sheep) guidelines [38]. Ideally, the animals were to be selected from the 2011 lamb crop and should represent a broad cross-section of the flock, not only those animals showing visible scour. A minimum of 20 animals were to be separated, individually marked by coloured spray-marker, the ear-tag number recorded and ten 50 ml universal sample pots were to be used to store ten faecal samples, one sample from each of 10 lambs. The samples were collected from the ground following defaecation after observation of the animals. For the post-treatment (pt) sampling, as many as possible of the initial ten lambs were to be resampled, although if it were not possible to collect from the initial ten, individuals from the remainder of the group were to be used as replacements [7]. Between sample times, the animals were to have full access to pasture already grazed that year. After collection, farmers were asked not to refrigerate samples, but to return them as soon as possible to the laboratory at VSD (AFBI), where the faecal egg counting was carried out.

Instructions on re-sampling times for the various drench classes were set as 7 days (d) following treatment, regardless of product selection. This follows SCOPS advice to “retest seven to 10 days (no longer, because of the short prepatent period of *N. battus*) after the administration of the drench.” [39, 40].

#### Sample processing

The sedimentation method for fluke eggs, as described by Flanagan et al. [41], was used for counting *Nematodirus* spp. eggs. Briefly, 3 g of faeces was added to 42 ml of water and homogenised, before passing the solution through a strainer and collecting a 15 ml sample in a test-tube. The sample was washed through a 90 μm sieve set over a 63 μm sieve, before inverting the 63 μm sieve and washing the filtrate into a bowl. The bowl contents were poured into a pint beaker and left to sediment for a minimum of 15 min. Eggs of *Nematodirus* spp. were counted and the number of eggs per gram (epg) was calculated. In order to check that all *Nematodirus* eggs were trapped and retained on the 63 μm sieve, the content remaining on the 90 μm sieve and the residue washed through the 63 μm sieve were regularly examined to confirm the absence of *Nematodirus* eggs. The detection limit of the method is 15 epg.

#### Faecal egg count reduction test (FECRT)

Percentage reduction was based on the formula of Kohapakdee et al. [42]: percentage reduction = [(T1 - T2)/T1] × 100, where T1 is the arithmetic mean Faecal Egg Count (FEC) pre-treatment and T2 is the arithmetic mean FEC of treated animals. This formula has been used in previous studies [7, 8, 43]. Resistance is confirmed when the reduction in FECs pt. is less than 95% and the lower 95% confidence interval of the percentage reduction is less than 90% [44].

#### Outlier identification

For the purposes of this investigation, an outlier was defined as a data point that was numerically distant from the remainder of points within said dataset [7]. If an individual FEC was outside the range of the arithmetic mean ± the standard error about the mean, it was not included in the efficacy calculation. Only pt. FECs were subject to outlier identification.

### Identification and initial characterization of ovine *Nematodirus* spp. present in Northern Ireland

*N. battus* eggs were identified in FECs on the basis of egg morphology [45], and this was subsequently verified on the basis of L_3_ morphology [46].

The evaluation of egg hatching behaviour followed the protocol of van Dijk and Morgan [19]. Embryonation was carried out at 20 °C. After 6 weeks, infective L_3_ larvae were visible in all eggs.

#### Species identification

Approximately 300 eggs were pipetted into a 2 ml Eppendorf tube and stored at 4 °C for 29 d, then transferred to a 13 °C incubator (Sanyo Incubator MIR-262; Sanyo Electric Biomedical Co. Ltd., Japan). At d 60, the eggs were stained in Lugol’s iodine for 20 min, counterstained in 30% (*w*/*v*) sodium thiosulphate, and then the hatched larvae were examined microscopically for species identification [46]. *N. battus* was the only *Nematodirus* species detected.

#### Hatching behaviour

Faecal material remaining after the FECRT analysis was pooled from all the farms and so the data represents the NI population as a whole. The eggs were split equally into two groups: one group was maintained at 4 °C for 29 d (the “Chilled” group), while the other was kept at 20 °C for the same time period (the “Non-Chilled” group). The groups were then transferred to a 13 °C incubator. Plates were redistributed daily so as to minimise any effect of temperature flux within the incubator. The proportions of eggs containing larvae, and any larvae that had hatched, were counted on d 0, and hatched larvae were thereafter counted on d 3 and on alternate days up to d 41. In order to determine whether the counts on d 41 represented the maximum hatch, further hatching was checked on d 50, 55 and 60.

## Results

### Questionnaire results

A total of 228 completed questionnaires (from the initial 252 distributed) were received. This represents a return rate of 90.5%. The summary of questions posed and responses received is presented in Table 1.

**Table 1:** The questions posed and potential responses to the questionnaire, together with the number (N) and percentage (%) of flock owners indicating the applicable response

Question	Potential Response	Percentage (number of respondents)
Do you treat specifically for *Nematodirus* spp. infection?	Yes No	81.6 (186) 18.4 (42)
How is the decision made to treat the animals?	AFBI forecast Faecal egg count Presence of scour Advice of veterinarian Advice of other farmers Same day annually Dependent on weather At turnout At 6 weeks of age As needed	19.7 (45) 7.9 (18) 47.4 (108) 14.5 (33) 53 (12) 23.7 (54) 6.6 (15) 1.3 (3) 5.3 (12) 5.3 (12)
Is every animal treated, or is only a percentage treated?	100% <100%	97.4 (222) 2.6 (6)
How is the volume of drench determined?	Estimate individual Group average Heaviest in group Weigh individual	40.3 (92) 10.2 (23) 48.2 (110) 1.3 (3)
Is the equipment checked before use?	Never Sometimes Always	17.3 (39) 52.0 (119) 30.7 (70)
Where is the product stored when not in use?	Fridge Cabinet/Cupboard Garage/Shed/Workshop	11.0 (25) 69.9 (159) 19.1 (44)
Is “best before” date checked before use?	Never Sometimes Always	8.0 (18) 29.3 (67) 62.7 (143)

(As the decision to treat is often multi-factorial, the denominator for percentage calculation is set as the number of returned questionnaires (228) and the numerator is the tally of flock owners indicating each potential response)

#### Treatment timing

The main influences on the decision of when to treat were: the presence of scour (47.4%), AFBI forecast warnings of the peak hatching of *Nematodirus* spp. eggs on pasture (19.7%), and the advice of veterinarians (14.5%), although 23.7% of flock owners treated on the same day each year regardless of the other factors named above (Table 1).

#### Potential under- or over-dosing

Dosing to the average weight of the group/flock was practised by 10.2% of respondents and the weight of the animals was estimated by 40.3% (Table 1), leading to the cumulative possibility of under- or over-dosing through incorrect weight estimation in 50.5% of flocks.

Ensuring that the equipment was delivering its stated dose before treatment was routinely practised by 30.7% of respondents, whilst 52.0% occasionally checked and 17.3% of respondents never checked their equipment before treating for *Nematodirus* spp. infection (Table 1).

Checking that the product was within its effective lifespan before use was routinely practised by 62.7% of respondents, occasionally checked by 29.3%, and never checked by 8.0% of respondents (Table 1).

#### Refugia

When asked whether every animal is treated or only a percentage of the flock is treated, 97.4% of flock owners indicated that every animal was given treatment for *Nematodirus* spp. infection (Table 1).

#### Product storage

Typically, the only storage requirements for anthelmintics are to store away from direct sunlight and within a temperature range of 4 °C – 25 °C. These conditions were satisfied by all respondents to the questionnaire: 69.9% of flock owners stored their anthelmintics in a cabinet or cupboard, 19.1% held them in either garage, shed or workshop, while the remainder (11.0%) chose to store their products in a refrigerator (Table 1).

#### Product use between 2009 and 2012

**Fig. 1:**
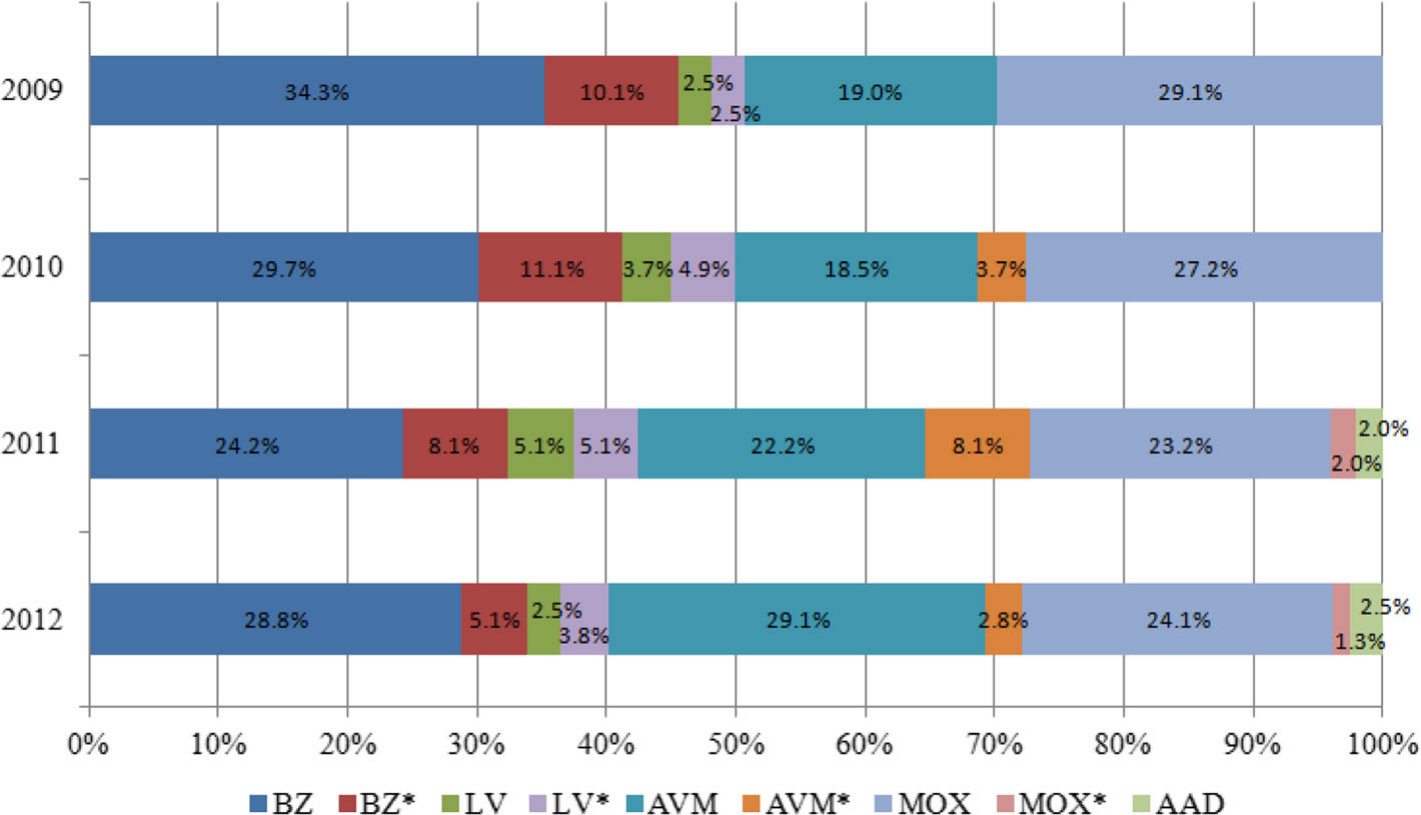
Product selection by anthelmintic group. AAD = amino-acetonitrile derivative; AVM = avermectin; BZ = benzimidazole; LV = levamisole; MOX = moxidectin; BZ*, LV*, AVM*, MOX* = part of a combination product (broad spectrum wormer plus flukicide)

The distribution of product use by product class over the 4-year time period is shown in Fig. 1. Over the surveyed period, BZ use (both as single active and in combination with a broad spectrum wormer) decreased from 44.4% in 2009 to 33.9% of all treatments given for nematodirosis in 2012. Similarly, the use of moxidectin (MOX) products fell from 29.1% to 25.4% of all treatments given between 2009 and 2012, while the use of avermectin (AVM) products increased by 12.9% over the same 4-year period. The amino-acetonitrile derivative (AAD), monepantel accounted for 2.0% and 2.5% of all treatments given in the Spring of 2011 and 2012, respectively. There was no observable trend for LV use, except that (excluding monepantel) it was the least frequently used anthelmintic for the control of *Nematodirus* spp. infection.

### FECRT

A total of 51 flock owners submitted pre-treatment faecal samples from their lambs in 2012. Of these, lambs from 41 farms had nematodirosis (as diagnosed by the presence of eggs) at the time of submission and sufficient volumes of faeces were submitted to allow the completion of the FECRT. The same 10 animals sampled at day 0 were sampled again at d 7 in 48 flocks. Within the remaining 3 flocks, only 9 of the initial 10 animals were sampled. In each case, the FEC of the replacement animal was out of character with the remainder of its dataset and was excluded from analysis. The pt. means for these 3 flocks were calculated from the remaining 9 animals. The influence that including these outliers within datasets had on efficacy calculations is summarised in Table 2.

**Table 2:** The impact of omitting outliers from mean post-treatment faecal egg counts on the efficacy of drug treatment

Flock code	AnthelminticGroup	Unadjusted		Corrected	
		Mean ± S.E.M.	Efficacy (%) (C.I.)	Mean ± S.E.M.	Efficacy (%) (C.I.)
1	AVM	5.3 ± 5.3	94 (48, 99)	0 ± 0	100 (100,100)
3	BZ	6.7 ± 4.9	98 (90, 99)	0 ± 0	100 (100,100)
10	BZ	30.3 ± 30.3	90 (11, 99)	0 ± 0	100 (100,100)

*AVM* avermectin, *BZ* benzimidazole, *C.I.* Confidence Intervals, *Corrected* outliers omitted from calculations, *S.E.M.* Standard Error about the Mean, *Unadjusted* with outliers included in calculations

**Fig. 2:**
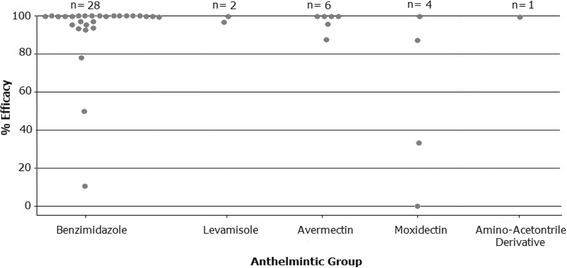
Efficacies (based on Faecal Egg Count Reduction, or FECR) following treatment for *Nematodirus battus* with the benzimidazoles, levamisole, the avermectins, moxidectin and the amino-acetonitrile derivative, monepantel

On the basis of observed treatment efficacy alone (Fig. 2), reduced efficacy of BZs was present in 35.7% (10/28) of flocks tested; of LV in 50% (1/2) of flocks tested; of AVMs in 33% (2/6) of flocks tested; and of MOX in 75% (3/4) of flocks tested. Reduced efficacy of AAD was not detected at the time of the survey.

### Hatching behaviour

**Fig. 3:**
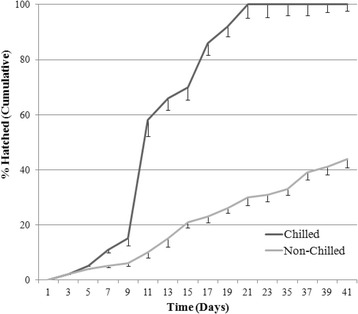
Hatching behaviour at 13 °C of *Nematodirus battus* eggs with or without prior chilling. The Figure represents the cumulative proportion of eggs hatched between day 0 and day 41. Three replicates were conducted per batch of eggs (error bars represent the standard deviation)

After a period of 41 days, 43% of eggs in the “Non-Chilled” group were able to hatch, and no further hatching was observed following d 50 (data not shown). Within the “Chilled” group, 100% of the eggs had hatched by d 21, with the largest single increase between time points occurring between d 9 and d 11 (Fig. 3).

## Discussion

The return rate for the Questionnaires was very high: 90.5%. They were distributed to sheep farmers who had participated in a previous survey and so were not randomly selected. This could be viewed as a limitation of the study. However, the farmers were respondents to an original, random distribution of Questionnaires to 1000 farmers in the province, and the sample size was of sufficiently large size to be meaningful, so this fact should not detract from the validity of the results. Moreover, it did allow us to monitor the same group of farmers with regard to the control of various helminth parasites: gastrointestinal nematodes, liver fluke and tapeworms [5, 8, 9, 47].

### Control of *Nematodirus*

There are a number of complex and confounding factors which influence the effectiveness of administered treatments. Broadly, they can be categorised as operational, chemical, parasitological and physiological. These areas are not mutually exclusive and many of the interactions between them play an important role in determining how effective a treatment will be at controlling the infection [48].

Adverse operational factors include treatment frequency, infrequent drug rotation, poor dosing technique, under-dosing and improperly timed treatments. Previous studies in NI have indicated that annual rotation between anthelmintics for control of ovine nematode parasites is practised by 21.3% of flock owners [8] and the majority (61.1% of all flock owners surveyed) apply treatments up to 2 times per year [8]. Although current guidelines advise that the volume of anthelmintic given to every animal should be that given to the heaviest of the group [38], potential under- or over-dosing was identified in the present study, as 40.3% of flock owners estimated the weight of the individual animal and a further 10.2% dosed all animals according to the average of the group. Therefore, potential under- or over-dosing through incorrect weight estimation exists in 50.5% of flocks in NI. Likewise, only 30.7% of owners checked their dosing equipment before treatment. It is recommended that flock owners incorporate local risk assessment (i.e. parasite forecasts) in their decisions regarding treatment timing to ensure optimum efficacy [39]. AFBI publishes an annual “*Nematodirus* forecast”, advising flock owners of the projected peak hatching of eggs. It is similar to the National Animal Disease Information Service (NADIS) forecast and to that of SCOPS, in that they all make use of meteorological data and the Ollerenshaw Index [49]. However, the AFBI forecast focuses solely on NI, offers advice on treatments, on efficacy testing should it be required, and provides a point of contact should flock owners have queries [50]. The questionnaire data revealed that only 19.7% of owners consulted the local AFBI forecast before treatments were given; similarly, only 6.6% of respondents took account of prevailing weather conditions. Generally, it is considered unwise to await the appearance of clinical signs before therapeutic intervention [51], although a total of 52.7% of flock owners did so.

Chemical factors contributing to treatment failure include the use of generic brands, expired products, improperly stored products and persistent anthelmintics. The Questionnaire returns indicated that generic products were available and used in NI, but the contribution of these to treatment failure has not been assessed. Over 60% of respondents indicated that they routinely ensured the product was within its “best before” date before use, although the potential also existed for uncontrolled storage temperatures of anthelmintics in fridges (11.0% of respondents) or in garages (19.1% of respondents). Due to their high safety index and efficacy [38], the BZs are widely used in the control of *N. battus* [37]. In this regard, the use of a BZ reduces selection pressure on the other anthelmintic families of drugs [39]. It might increase non-target pressure on other nematode species that might be present at the time of treatment, but BZ resistance is so widespread in these species that use against *N. battus* is unlikely to exacerbate significantly the (already irretrievable) situation. Furthermore, the timing of BZ use in Spring for lambs is likely to be too early to have an impact on the other PGE species. Over the time period investigated by the questionnaire, the use of AVMs for *Nematodirus* spp. control increased by 12.9% (to 31.9%: Fig. 1). This is an important point, as MLs (AVMs plus MOX) (57.3% in 2012) are used more extensively than BZs (33.9%); LV (6.3%) and AAD (2.5%) are not widely used and so are not major contributors to the potential problem of inappropriate drug choice.

### Ineffective control of *Nematodirus* in NI

In *Nematodirus* spp., testing the efficacy of treatments using FECRT should, where possible, have pt. sampling occurring within 7–10 d following drench administration [39]. This is due to the brief pre-patent period of *N. battus* [52]. The World Association for the Advancement of Veterinary Parasitology (WAAVP) guidelines recommend pre-dosing egg counts of at least 150 epg for the faecal egg count reduction test to give reliable evidence of resistance (based on the modified McMaster technique with minimum sensitivity of 50 epg). This is clearly difficult to obtain for *Nematodirus* spp., for which egg counts are often low and the presence or absence of even a single egg in the small sample examined could lead to over- or under-estimation of the egg count, respectively. Hence, if evidence of resistance is to be obtained, a more sensitive assay than the standard McMaster is needed. The sedimentation method allows a much larger sample to be analysed, leading to a more accurate estimation of the number of eggs present. Regular and repeated checks on the content of the 90 μm sieve and on the residue passing through the 63 μm sieve following sample preparation, have confirmed the efficacy of the method for retention of *Nematodirus* eggs and, with a detection limit of 15 epg, it is more sensitive than the modified McMaster method for the low egg counts typically found in *Nematodirus* infections. Other techniques now exist with greater sensitivity than the McMaster technique - eg FLOTAC, 1 epg [53] and mini-FLOTAC, 5 epg [54] - but they were not available to use at the time the present study was carried out.

While at first glance it would appear that there are a high number of holdings in NI where reduced drug efficacy is a problem, this may not in fact be the case. The five classes of anthelmintics claim efficacy against *N. battus* [55], although the parasite is a dose-limiting species for mebendazole (and other early, insoluble BZs) [27] and for most of the ML anthelmintics [37]. Reduced BZ activity was present in 35.7% of flocks tested with BZs. Potentially, this constitutes a significant problem, as the BZs are the main class of drugs used for control of nematodirosis.

Four flock owners used products containing MOX. Injectable formulations of MOX are only active against *N. spathiger*, whereas oral MOX is active against *N. battus* (and other *Nematodirus* spp). Three of the four flock owners used oral MOX; the other used injectable, long-acting MOX. In the case of the AVMs, Dectomax, Noromectin, Oramec and Qualimec were used. Dectomax® (Zoetis) will only effectively treat L_4_ *N. battus* larvae when a higher dose rate (of 300 μg/kg, not 200 μg/kg) than that suggested on the packaging is used. (When contacted, the flock owner did not know this.) Ivomec® (Merial AH) and Qualimec® only have a label claim against *N. filicollis* [55]. Thus, it would appear that the use of ML products to control nematodirosis should not be recommended until flock owners are more aware of the gaps in their spectra of activity.

The presence of reduced drug efficacy against *N. battus* infection in NI has highlighted a major issue and illustrates the problem that farmers have to deal with. Significantly, it reflects the reality of what is happening in the field. One possible explanation for this state of affairs is the existence of AR in *N. battus* populations, but that has not been demonstrated in the present study.

### Anthelmintic resistance in Nematodirus spp., across the British isles and the ROI

Results of a survey conducted in the ROI in 2013 revealed reduced treatment efficacy against *Nematodirus* spp. While BZs were effective in 100% of tests, LV was effective in 80% of cases and ML (AVM plus MOX) treatment was effective in 94% of cases [34]. No details were given regarding which ML products were used, but the authors noted that *Nematodirus* spp. is the dose-limiting species for that anthelmintic group and this may account for the lower efficacy of MLs in some cases.

In a preliminary investigation into a case of suspected BZ resistance in *N. battus*, in the North of England, it was shown that treatment with fenbendazole reduced the FEC by 83% [36]. Faecal material from the farm with suspected BZ resistance was subsequently used to generate parasite material for a clinical efficacy trial, which confirmed the presence of AR [37].

### Hatching requirements

Within-genotype variation has been put forward as a hypothesis regarding the diversity of hatching requirements observed in a similar study in the UK [20]. This represents a viable hypothesis as, in the present study, any eggs collected throughout the Spring infection would be the offspring of larvae which had hatched after a chilling stimulus; the good weather conditions (mean daily temperatures of 13.5 °C, 13.3 °C and 11.1 °C in August, September and October, respectively) for hatching in the Autumn months preceding sample collection in the Spring of 2012 precluded the prospect that the Spring infections arose from eggs not requiring chilling (i.e. those which had been unable to hatch during the previous Autumn); and larvae from eggs hatching in Autumn would not have survived the winter. As stated by van Dijk and Morgan [20], “The eggs collected for the experiments, therefore, must have been produced by nematodes hatching from eggs that had delayed hatching until after exposure to a chilling stimulus”.

Previously, we have discussed the shift in focus away from the classical Spring bell-curve of *Nematodirus* spp. infection towards Autumn infections. This shift was consistent with increasing monthly temperatures [4]. How the incidence of one affects the incidence of the other is not yet clear, but some reports suggest a positive correlation between the heights of the Spring and Autumn peaks of diagnoses within the same year [56]. While Autumn hatching and *N. battus* infection does occur in NI, probably it is not a disease entity in its own right, but would occur as an element of PGE at that time. The animals are older and larger, and more resilient, so the clinical problems (of diarrhoea and death) are not likely to be as severe as spring nematodirosis.

## Conclusions

The results of the questionnaire survey on control of *Nematodirus* identified a number of poor control strategies, namely, inappropriate product choice, under-dosing and inaccurate calibration of dosing equipment. This highlights the need for continued education of flock owners and a greater awareness by them of the range of efficacies of those products that are available. An unsuitable choice of anthelmintic has implications, not just for control of *Nematodirus*, but for the development of AR in off-target species as well. In the short-term, the use of BZs over AVMs should be promoted for the control of nematodirosis, in order to reduce the pressure on the AVM group. Stockholders should also make better use of forecasting information and show greater vigilance in monitoring parasitic infections. They should be encouraged to submit samples for diagnosis to ensure optimal treatment timing and detection of AR. The early detection of AR would allow maximal time to implement effective control strategies aimed at reducing further development of resistance [57]. The results of the coprological survey have highlighted the problem of reduced activity of anthelmintics used to treat *Nematodirus* infections and this may indicate that AR exists in *Nematodirus* populations in NI. The latter requires more rigorous investigation before it can be confirmed. This is particularly true for LV, AVM and MOX, as only a small number of farms were involved in the present study and so the results should be interpreted with some degree of caution.

The results obtained in the hatching study suggest that a diversity of behaviours exists. This may indicate within-genotype variation in the chilling requirement and, as such, further investigations into the long-term effects of climate change on parasitism, flock health plans and anthelmintic use are warranted. The existence of a second autumnal infection, and its importance relative to the Spring infection, requires ongoing monitoring and is something farmers should be aware of. Clearly, signs of scour in the autumn would not necessarily alert farmers to the possibility of *N. battus* infection and they would most likely be using MLs, not BZs, because of the need to treat other nematode species (at that time). Consequently, accurate diagnosis of *N. battus* at this time is essential, as is better education of farmers to be more aware of an autumnal infection.

If appropriate measures are put in place, AR should not develop in *Nematodirus* spp. to the same extent as already reported for *Trichostrongylus* spp. and *T. circumcincta* in NI [7]. While *Nematodirus* spp. may not be seen to be as significant as these species, its successful control is still of great importance to the health of young lambs in the flock.

Finally, while the present communication completes the overview of ovine helminth disease in NI, it contributes to a greater all-island of Ireland perspective on disease control in Ireland. The data may help to inform the design and implementation of future management programmes.

## Abbreviations

AAD: Amino-acetonitrile derivative
AFBI: Agri-Food and Biosciences Institute
AR: Anthelmintic resistance
AVM: Avermectin
BZ: Benzimidazole
CAFRE: College of Agriculture, Food and Rural Enterprise
DAERA: Department of Agriculture, Environment and Rural Affairs
DAFM: Department of Agriculture, Food and the Marine
FEC: Faecal Egg Count
FECRT: Faecal Egg Count Reduction Test
GVA: Gross added value
LV: Levamisole
MAFF: Ministry of Agriculture, Fisheries and Food
ML: Macrocyclic lactone
MOX: Moxidectin
NADIS: National Animal Disease Information Service
NI: Northern Ireland
NIFDA: Northern Ireland Food and Drink Association
NOAH: National Office of Animal Health
PGE: Parasitic gastroenteritis
pt.: Post-treatment
ROI: Republic of Ireland
SAC: Scottish Agricultural College
SCOPS: Sustainable Control of Parasites in Sheep
SNP: Single nucleotide polymorphism
UK: United Kingdom
VSD: Veterinary Sciences Division
WAAVP: World Association for the Advancement of Veterinary Parasitology
